# Clinical efficacy and mechanisms of hyperbaric oxygen therapy in the treatment of rheumatic and immune diseases

**DOI:** 10.3389/fmed.2025.1706637

**Published:** 2025-12-18

**Authors:** Jinying Fang, Wei Li, Chunping Liu, Yonghong Wang, Jie Hu, Qinglu Sun, Hailong Wang

**Affiliations:** Dongzhimen Hospital, Beijing University of Chinese Medicine, Beijing, China

**Keywords:** rheumatic and immune diseases, hyperbaric oxygen therapy, fibromyalgia syndrome, vasculitis, systemic sclerosis

## Abstract

Rheumatic and autoimmune diseases represent one of the major causes of chronic joint and muscle pain, skin ulceration, and mental depression, significantly impairing patients' physical and psychological wellbeing as well as their quality of life. Current evidence suggests that hypoxia may play a role in the pathogenesis and progression of rheumatic and autoimmune diseases and their associated complications. Hypoxia can induce pathological cellular stress, thereby triggering cell death. Hyperbaric oxygen therapy (HBOT) is a well-established, effective, and safe method for significantly increasing dissolved oxygen content in plasma and arterial oxygen partial pressure. Based on a comprehensive review of all relevant literature published in the past decade and indexed in PubMed regarding HBOT for rheumatic and autoimmune diseases, the following findings were observed: HBOT demonstrated an efficacy rate of 87.5%−100% in treating rheumatic and autoimmune diseases complicated by skin ulcers. For patients with fibromyalgia syndrome (FMS), the pain relief rate ranged from 87.5 to 100%. Additionally, HBOT exhibited favorable therapeutic effects in cases involving sensorineural hearing loss and acute macular neuroretinopathy secondary to rheumatic and autoimmune diseases. Regarding safety, adverse effects were reported in seven studies, primarily including mild barotrauma, tinnitus, headache, and claustrophobia. All adverse events resolved upon discontinuation of HBOT, and no severe adverse reactions were documented.

## 1 Introduction

Hyperbaric Oxygen Therapy (HBOT) is a non-invasive treatment modality in which patients breathe 100% oxygen under conditions exceeding 1 absolute atmosphere (ATA) of pressure. Initially employed in the 1930s for decompression sickness (1), HBOT has since evolved into an established therapeutic approach for a variety of conditions, including non-healing wounds, infections, and medical emergencies, as well as an adjunctive treatment option for certain inflammatory disorders (2, 3). Oxygen plays a pivotal role in numerous physiological processes, reaching all tissues and cells via systemic circulation. By creating a high-pressure, oxygen-enriched environment, HBOT significantly enhances dissolved oxygen levels in plasma and arterial oxygen partial pressure. Consequently, HBOT exerts broad effects on cellular biochemical and physiological responses, including promoting angiogenesis, mitigating localized inflammatory reactions, and counteracting oxidative stress (4–6). HBOT addresses the ischemia, hypoxia, and chronic inflammation common in many diseases through its distinct physiological mechanisms, thereby promoting tissue repair and alleviating symptoms. It does not replace conventional immunosuppressants or biologics but acts as a complementary approach to improve overall treatment efficacy (6).

Rheumatic immune diseases (RIDs) can lead to joint and muscle pain, functional impairment, and clinical manifestations such as skin ulceration and sudden sensorineural hearing loss, severely impacting patients' physical and mental health as well as quality of life. Current pharmacologic treatments for these conditions primarily include corticosteroids, immunosuppressants, biologic agents, and non-steroidal anti-inflammatory drugs (NSAIDs). However, some patients fail to achieve satisfactory clinical outcomes (7). It is widely recognized that numerous rheumatic and autoimmune diseases—such as vasculitis, fibromyalgia, and systemic sclerosis—are closely associated with aberrant immune-inflammatory responses and oxidative stress (8). Local hypoxia can trigger physiological and pathological alterations, including oxidative stress, angiogenesis, vascular remodeling, inflammatory responses, and metabolic reprogramming, thereby driving disease progression (6, 9). Thus, hypoxia may play a critical role in the pathogenesis of rheumatic and autoimmune diseases, and HBOT may demonstrate therapeutic efficacy against these conditions and their complications.

As a novel therapeutic strategy for RIDs, HBOT offers distinct advantages, including its non-invasive nature, favorable safety profile, and demonstrated efficacy in alleviating common clinical symptoms across diverse patient populations. This review aims to summarize the therapeutic effects, safety, and underlying mechanisms of HBOT in the management of rheumatic and autoimmune diseases, thereby providing a foundation for future clinical and basic research.

## 2 Methods

### 2.1 Data sources and searches

We performed electronic searches using exploded Medical Subject Headings (MeSH) terms and various keyword combinations. The search terms included MeSH exp “Hyperbaric Oxygen Therapy” along with the keywords “Hyperbaric Oxygenations,” “Oxygenations, Hyperbaric,” “Hyperbaric Oxygen Therapies,” “Oxygen Therapies, Hyperbaric,” “Oxygen Therapy, Hyperbaric,” “Therapies, Hyperbaric Oxygen,” “Therapy, Hyperbaric Oxygen,” “Oxygenation, Hyperbaric,” “rheumatic immune diseases,” “Rheumatic and immune diseases,” “Fibromyalgia syndrome,” “Vasculitis,” “thromboangiitis obliterans,” “Behçet's disease,” “systemic lupus erythematosus,” “rheumatoid arthritis,” “fibromyalgia syndrome,” and “systemic sclerosis.” We also manually examined the reference lists of included textbooks, retrieved studies, review articles, and academic congress reports. The referenced research articles were obtained from PubMed.

### 2.2 Inclusion criteria and exclusion criteria

#### 2.2.1 Inclusion criteria

(1) Clinical research studying the treatment of rheumatic immune diseases with HBOT, published in either English language; (2) the participants were defined as those diagnosed with rheumatic immune diseases such as vasculitis, fibromyalgia syndrome, systemic lupus erythematosus, etc.; and (3) the types of studies included encompass case reports, case series, cohort studies, and controlled trials.

#### 2.2.2 Exclusion criteria

(1) Patients with other severe diseases that could influence the outcomes, such as severe heart failure, cancer, DIC, severe infection; (2) studies that were abstracts, reviews, comments, and editorials, etc.; and (3) literature with repetitive content.

## 3 Results

### 3.1 Clinical applications and research advances of HBOT

Oxygen is essential for aerobic respiration in human cells, a process that occurs in the mitochondria, where approximately 80% of oxygen is consumed, while the remaining 20% is utilized by other organelles. Hypoxia, defined as a decrease in tissue oxygen tension, induces cellular pathological stress and is closely associated with the onset and progression of various diseases, such as acute kidney damage (10), myocardial infarction (11), and neurological injury (12). Hypoxia leads to increased oxidative stress, resulting in the generation of reactive oxygen and nitrogen free radicals. These radicals are highly cytotoxic, causing cellular damage and ultimately inducing cell death.

Hypoxia-inducible factor 1 (HIF-1) serves as a key regulator of metabolic reprogramming in hypoxic cells (13, 14). HIF-1 modulates critical physiological and pathological responses to hypoxia, including oxidative stress, angiogenesis, vascular remodeling, and inflammatory reactions. Furthermore, HIF-1 regulates the proliferation, migration, oxidative stress response, immune function, and cell death of various core cell types, such as cardiac cells, endothelial cells, smooth muscle cells, and macrophages (15).

HBOT ameliorates hypoxia by enhancing oxygen delivery and suppressing HIF-1 activity, thereby reducing oxidative stress, promoting tissue repair, enhancing vasoconstriction and angiogenesis, and attenuating local inflammation. These mechanisms collectively influence both physiological and pathological cellular responses in the human body (16).

### 3.2 Application of HBOT in RIDs

**Figure 1:**
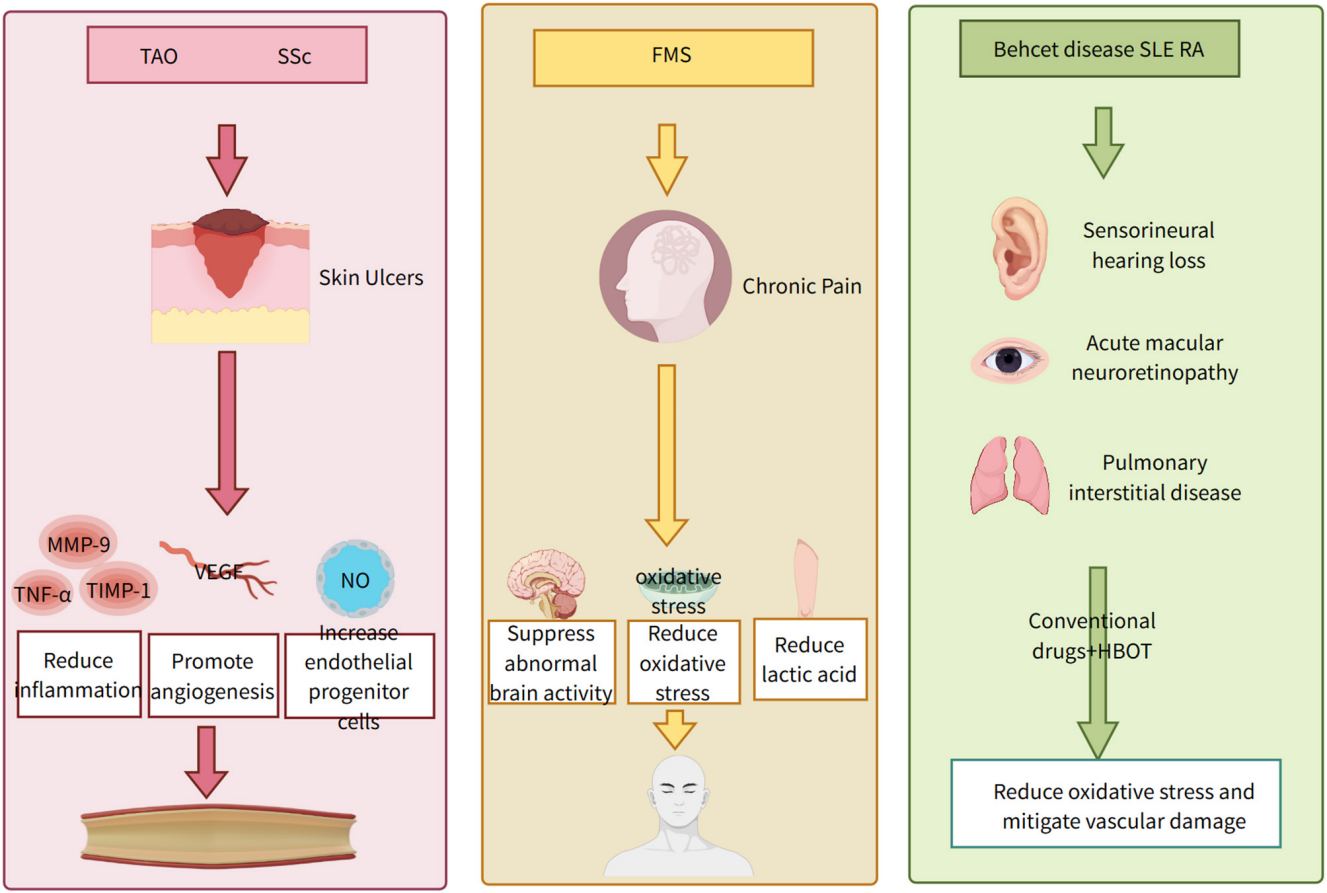
Dosage, duration and mechanisms of HOBT in different rheumatic and immunological disease complications.

In recent years, an increasing number of clinical reports have documented the use of HBOT for treating various conditions, including skin injuries (17), neurodegenerative diseases (18, 19), sudden sensorineural hearing loss (20), and aging-related disorders (21). However, the application of HBOT in rheumatic immune diseases remains relatively limited. This article reviews all PubMed-published literature from the past decade on HBOT for rheumatic immune diseases, encompassing 21 studies involving 343 patients with conditions such as vasculitis, fibromyalgia syndrome (FMS), and systemic sclerosis (SSc). These findings suggest that HBOT represents a novel therapeutic approach for rheumatic immune diseases, demonstrating promising efficacy and unique advantages, as summarized in Table 1 and Figure 1.

**Table 1:** Relevant information from literature on HBOT for rheumatologic and immune diseases.

**Paper**	**Study type**	**Sample**		**Disease**	**Complication**	**Treatment**		**Number of HBOT Sessions**	**Treatment Cycle (w)**	**Follow-up (m)**	**Observation indicators**	**Outcome**	**Adverse reactions**		**Drop out**
		**Treatment Group**	**Control Group**			**Treatment Group**	**Control Group**						**Treatment Group**	**Control Group**	
Lee et al. (2023) (53)	Case Report	1	—	LV	Painful Ulcer	120 min, 2 ATA, 3 d/w	—	13 times	4	8	—	Skin ulcers healed, with no recurrence during follow-up	—	—	—
Pathault et al. (2024) (54)	Retrospective Study	18	—	a	Painful Ulcer	—	—		1–48		—	Pain was significantly reduced in 83.3% of patients, strong opioid use was significantly decreased, and local ulcers improved	—	—	—
Herrera-Sánchez et al. (2022) (55)	Case Report	1	—	LV	Skin ulcer	60 min, 1.45 ATA	conventional drugs	12 times		6	—	Ulcer healing | Ulcer healed completely, with no recurrence	—	—	—
Mirasoglu et al. (2017) (25)	Retrospective Study	6	—	SSc	Skin ulcer	90 min, 1.45 ATA	—	14 times	6		—	Complete healing was observed in 4 patients, near-complete healing in 2 patients; no amputations were required	—	—	—
Biney et al. (2020) (26)	Case Report	1	—	SSc	Skin ulcer?PAH	90 min, 2 ATA	conventional drugs	30 times		22	—	Wound healed; cardiac function was at WHO Class II	—	—	—
Hemsinli et al. (2018) (24)	Retrospective Study	47	50	TAO	Skin ulcer	90 min, 2.37 ATA, 5 d/w	conventional drugs			30	—	21 patients in the HBOT group were healed at 10 months; the number of major amputations was significantly lower, and VAS scores were reduced	?	?	—
Hemsinli et al. (2016) (56)	Retrospective Study	36	—	TAO	Skin ulcer	—	—	—	—	—	VAS	52.7% of patients recovered completely	—	—	—
Chuang et al. (2024) (57)	Case Report	1	—	BD	SNHL	—	Oral Prednisolone, Intratympanic Steroid Injection	15 times	—	—	—	Hearing threshold improved by 20 dB, and speech recognition threshold improved	—	—	—
Shroff et al. (2023) (50)	Case Report	2	—	SLE	AMN	—	High-Dose Steroid Therapy	12 times	—	12	—	No significant retinal thinning was observed on optical coherence tomography. Complete resolution of the visual field scotoma was noted	—	—	—
Nakatani et al. (2017) (52)	Case Report	1	—	GPA	Pneumatosis intestinalis	90 min, 2 ATA	Conventional Medication	13 times	2.5	36	—	No recurrence was observed during the 3-year follow-up	?	?	—
Hadanny et al. (2018) (58)	RCT	15	15	FMS	CSA	90 min, 2 ATA, 5 d/w	Psychotherapy	60 times	12		—	b	One patient reported headache, and 12 experienced mild barotrauma	None	——
Atzeni et al. (2019) (59)	Prospective Study	32	—	FMS		90 min, 2.5 ATA, 3 d/w	—	20 times	4	—	—	Significant improvements were observed in pain scores, anxiety symptoms, fatigue, and FM symptom severity scores; no significant change was noted in sleep quality	Two patients developed MEBT, one reported claustrophobia, one experienced dizziness, and one reported drowsiness	—	Treatment was discontinued in two patients due to MEBT, in one due to claustrophobia, and in one due to dizziness
Efrati et al. (2015) (60)	RCT	27	26	FMS		90 min, 2 ATA, 5 d/w	None	40 times	8	—	—	FM symptoms and quality of life were significantly improved in the HBOT group; abnormal brain activity was corrected	Five patients experienced dizziness, claustrophobia, and inability to equalize ear pressure; 13 patients had mild barotrauma	—	Five patients decided to discontinue HBOT due to dizziness, claustrophobia, and inability to equalize ear pressure
Izquierdo-Alventosa et al. (2020) (40)	RCT	17	16	FMS	—	90 min, 1.45 ATA, 5 d/w	Standard Care	40 times	8	—	—	Pressure pain threshold, endurance, functional capacity, and physical function were increased; fatigue and pain perception were significantly improved	–	—	—
Guggino et al. (2020) (61)	RCT	22	14	FMS	—	90 min, 2 ATA, 5 d/w	None	40 times	8	—	Number of tender points, Pain VAS, Fatigue VAS, WPI, FACIT fatigue, PSQI	—	–	—	—
Curtis et al. (2021) (38)	Prospective Study	9	8	FMS	—	90 min, 2 ATA, 5 d/w	Standard Care	40 times	8	3	FIQR, HADS, FSS, JSS, PGIC	Global function, anxiety and depression symptoms, and sleep quality were significantly improved in the HBOT group	mild barotrauma of the middle ear (three patients), new-onset myopia (four patients)	—	—
Casale et al. (2019) (62)	系列报道	25	–	FMS	—	90 min, 2.4 ATA	–	20 times	4		NME	NME was significantly increased; no changes were observed in maximal force, EMG amplitude, or muscle fiber CV		—	—
Bosco et al. (2019) (63)	Prospective Study	12	–	FMS	Interstitial cystitis	90 min, 2 ATA, 5 d/w	–	20 times	4		WPI	Cystoscopy revealed reduced petechiae and resolution of Hunner's ulcers	–	—	—
Rahav Boussi-Gross et al. (2024) (39)	RCT	24	24	FMS	CSA	90 min, 2 ATA	Standard Care	60 times	12	—	FIQS, WPI, SSS, BSI, PSS, BDI, MSDQ, SF-36, SPECT	All subjects were superior in the HBOT group compared to the control group	Mild barotitis media (seven patients), emotional distress (three patients), childhood trauma (two patients), temporary blurred vision (one patient)	c	In the control group, 14 participants discontinued the treatment prior to the end of the 12-week period due to side effects, whereas 8% of the treatment group withdrew early
Izquierdo-Alventosa et al. (2024) (37)	RCT	17	16	FMS	—	90 min, 1.45 ATA, 5 d/w	Standard Care	40 times	8	—	VAS, PCS, CPAQ, PIPS, PSPS, SF-12	Pain-related indicators, quality of life | Indicators related to pain and quality of life were significantly improved in the HBOT group compared to the control group	—	—	—
Ablin et al. (2023) (64)	RCT	29	29	FMS	—	90 min, 2 ATA, 5 d/w	Standard Care	60 times	12	—	VAS, WPI, SF-36, MOS, BSI-18, PPTSPECT	FM pain indicators, quality of life, mood, and social function were improved in the treatment group; increased brain activity was observed in the frontal and parietal regions	Mild barotitis media (13 patients), headache (1), 1 transient blurred vision (1), tinnitus (2), allergic reaction (1)	d	e

ATA, Atmosphere Absolute; SSc, Systemic Sclerosis; LV, Livedoid Vasculopathy; FM, Fibromyalgia; TAO, Thromboangiitis Obliterans; GPA, Granulomatosis with Polyangiitis; SNHL, Sensorineural Hearing Loss; RCT, Randomized Controlled Trial; BD, Behcet disease; min, minute(s); d, day(s); w, week(s); m, month(s); MEBT, Middle Ear Barotrauma that can be Eliminated; CSA, Childhood Sexual Abuse; AMN, Acute Macular Neuroretinopathy; SLE, Systemic Lupus Erythematosus; FIQR, Revised Fibromyalgia Impact Questionnaire; HADS, Hospital Anxiety and Depression Scale; FSS, Fatigue Severity Scale; JSS, Jenkins Sleep Scale; PGIC, Patient Global Impression of Change; WPI, Widespread Pain Index; SSS, Symptom Severity Score; BSI, Brief Symptom Inventory; PSS, PTSD Symptom Scale; BDI, Beck Depression Inventory; MSDQ, Multiscale Dissociation Inventory; SF-36, RAND 36-Item Health Survey; VAS, Visual Analog Scale; PCS, Pain Catastrophizing Scale; CPAQ, Chronic Pain Acceptance Questionnaire (Short Form); PIPS, Pain Intensity Scale; PSPS, Personal and Social Performance Scale; SF-12, 12-Item Short Form Health Survey; WPI, Widespread Pain Index; MOS, Medical Outcomes Study Sleep Scale; BSI-18, Brief Symptom Inventory-18; PPT, Pressure Pain Threshold assessed by a handheld computerized pressure algometer; SPECT, Single Photon Emission Computed Tomography.

a: Calciphylaxis (*n* = 6), vasculitis (*n* = 4), leg ulcers (*n* = 3), mixed ulcers (*n* = 3), thromboangiitis obliterans (*n* = 1), and LV (*n* = 1).

b: The HBOT group demonstrated significant improvements in FMS questionnaire scores, quality of life domains, PTSD symptoms, and psychological distress. Brain SPECT imaging revealed significantly increased brain activity in the prefrontal cortex, orbitofrontal cortex, and subgenual areas, with all improvements being more pronounced compared to the control group.

c: Increased fatigue (37%), increased pain (29%), increased emotional distress (25%), and digestive/stomach pain (20%). Other adverse effects included weakness and fainting, weight gain, headache, limb numbness, and sleep disturbances.

d: Dizziness (58.6%), drowsiness/weakness (20.7%), nausea (13.8%), and increased pain (10.3%). Other adverse effects included lack of improvement, deterioration, dry mouth, palpitations, headache, and allergic reactions.

e: Two patients in the HBOT group were excluded, one due to a concurrent illness and one due to poor compliance who did not complete the post-protocol assessment. Two additional patients withdrew consent during the treatment period and did not complete the assessments. In the medication group, two patients were excluded due to poor compliance and failure to complete the follow-up assessments.

#### 3.2.1 Efficacy and mechanisms of HBOT in treating skin ulcers associated with RIDs

Since tissue regeneration in wounds requires oxygen, exposure to 100% oxygen accelerates this process. HBOT has been applied in traumatic wounds, thermal burns, calciphylaxis, skin grafts, radiation-induced injuries, diabetic ulcers, and other wound types.

In rheumatic immune diseases, HBOT is frequently utilized for vasculitis-related skin ulcers. Vasculitis, characterized by chronic inflammation of the vascular wall and surrounding tissues, can lead to vascular and organ damage. Its pathological manifestations primarily include collagen fiber degeneration, fibrin deposition, and endothelial cell necrosis (22, 23). Skin ulcers in patients with thromboangiitis obliterans (TAO) are associated with amputation and mortality. A retrospective study involving 97 TAO patients with Fontaine stage III ischemic wounds (47 in the HBOT group and 50 in the conventional treatment group) demonstrated that the HBOT group exhibited a significantly lower major amputation rate after 10 months of treatment (2/47 vs. 13/50, *P* = 0.007). Compared to conventional therapy, the HBOT group showed significant improvements in the number of patients regressing to Fontaine stage I (27/47 vs. 17/50, *P* = 0.035), complete wound healing (21 vs. 11, *P* = 0.031), and Visual Analog Scale (VAS) scores (*P* < 0.001). The addition of HBOT to standard treatment for TAO patients with non-healing ischemic wounds and severe limb pain provided substantial benefits in wound healing and pain resolution (24).

Skin ulcers are also a common complication of SSc, significantly impairing quality of life. A retrospective study of six SSc patients with ulcers treated with HBOT revealed that all patients had undergone at least 6 weeks of conventional therapy with poor outcomes. Following HBOT, four patients achieved complete ulcer resolution, while two exhibited near-complete healing (25). Pulmonary arterial hypertension (PAH), another life-threatening complication of SSc, has unclear responsiveness to HBOT. A case report described a 65-year-old female SSc patient with severe PAH and a venous ulcer in the left lower limb. After adjunctive HBOT, the ulcer resolved completely, quality of life improved, and cardiopulmonary function remained stable without adverse effects (26). Additionally, HBOT has been reported to aid in treating chronic skin ulcers associated with IgG4-related skin disease (27).

Based on existing clinical studies, 83.3%−100% of rheumatic immune disease patients with skin ulcers benefited from HBOT, with 44.68%−100% achieving complete ulcer resolution and no recurrence during follow-up.

HBOT also upregulates host factors such as tumor necrosis factor-α (TNF-α), matrix metallopeptidase 9 (MMP-9), and tissue inhibitor of metalloproteinase-1 (TIMP-1), thereby mitigating local inflammatory responses (28, 29). Several studies indicate that HBOT significantly promotes angiogenesis while reducing inflammation. By elevating vascular endothelial growth factor (VEGF) levels, HBOT enhances angiogenesis in injured tissues, facilitating wound healing (4). Furthermore, HBOT increases nitric oxide levels, augments endothelial progenitor cell populations, upregulates epithelial growth factors and angiogenesis-related proteins, and downregulates apoptosis-associated proteins, thereby promoting re-epithelialization, endothelial cell migration, and granulation tissue formation (30).

Animal studies comparing normobaric hyperoxia therapy (NBOT) with HBOT reaffirmed that higher oxygen pressures are required to induce angiogenesis (31). *In vivo* experiments demonstrated that HBOT enhances stem cell proliferation in intestinal crypts and stimulates angiogenesis in the chick embryo chorioallantoic membrane (32). In a clinical trial involving patients with chronic non-healing wounds (unresolved for over 20 months), standardized HBOT (20 sessions, five sessions/week) elevated VEGF and interleukin-6 levels while reducing endothelin-1 levels. These findings suggest that HBOT creates a hyperoxic environment that activates host-derived wound resolution and angiogenic factors, thereby promoting vascularization (33).

In summary, HBOT attenuates local inflammation, accelerates angiogenesis, reduces lesion size, improves wound healing rates, and significantly decreases amputation risk.

#### 3.2.2 Efficacy and mechanisms of HBOT in treating chronic pain in RIDs

Chronic pain is defined as pain persisting for more than 3 months, which can lead to depression and suicide, affecting over 30% of the global population (34). It can be classified as nociceptive, neuropathic, or nociplastic. Nociplastic pain differs mechanistically from the other two types and is generally associated with enhanced pain and sensory processing in the central nervous system, as well as altered pain modulation. Nociplastic pain may occur independently, often in conditions such as FMS (35). FMS is a syndrome characterized by chronic widespread pain and a range of other somatic and psychological manifestations, significantly impairing patients' quality of life (36).

HBOT may improve joint and muscle pain, fatigue, and sleep disturbances in FMS patients (37–39). A randomized controlled trial (RCT) involving 33 FMS patients divided into an HBOT group (*n* = 17) and a control group (*n* = 16) assessed induced fatigue, perceived pain, pressure pain thresholds, endurance and functional capacity, physical performance, and cortical excitability. The results demonstrated that, compared to the control group, the HBOT group exhibited significant improvements in pressure pain thresholds, endurance and functional capacity, and physical performance (*P* < 0.05). Additionally, HBOT increased pressure pain thresholds, endurance and functional capacity, and physical performance, while also significantly alleviating induced fatigue and resting pain perception (*P* < 0.05) (40). No adverse effects were observed during treatment. Current clinical studies indicate that 87.5%−100% of FMS patients benefit from HBOT, with marked reductions in pain and fatigue scores. Adverse effects, such as mild barotrauma-induced otitis media, headache, tinnitus, and claustrophobia, were transient and resolved without serious complications.

A meta-analysis incorporating nine studies with a total of 288 patients (185 receiving HBOT) found that HBOT significantly alleviated pain in FMS patients compared to the control group (*P* < 0.001). Most included studies reported that HBOT improved tender points, fatigue, and sleep disturbances in FMS. Among HBOT-treated patients, 44 (23.8%) experienced adverse events, and 12 (6.5%) discontinued treatment due to side effects, with no serious adverse events reported. Lower pressure (less than 2.0 ATA) may reduce adverse events in FMS (41).

The pathogenesis of FMS remains unclear, though studies suggest abnormal brain activity in pain-related regions in FMS patients (42), which may contribute to chronic pain. HBOT reduces brain activity in the posterior cortex while increasing activity in the frontal lobe, cingulate gyrus, medial temporal lobe, and cerebellar cortex, thereby inducing beneficial changes in functionally aberrant brain regions and improving symptoms and quality of life in FMS patients (43). Another hypothesis posits that localized hypoxia may induce muscular changes leading to chronic pain and reduced lactate concentrations (42). Oxidative stress may play a pivotal role in FMS pathophysiology, with elevated levels of nitric oxide, lipid peroxidation, and mitophagy contributing to pain hypersensitivity (44). A promising mechanism of HBOT is its ability to mitigate oxidative stress under hypoxic conditions. This therapy inactivates caspase-3 and caspase-9 while upregulating the expression of the apoptosis-regulating gene Bcl-2 (45), suggesting that hyperbaric oxygen therapy enhances cellular oxygen availability, reduces mitochondrial-induced apoptosis, and preserves mitochondrial function. Reduced inflammatory cytokines may further promote comprehensive functional improvement and pain relief in FMS patients (44).

#### 3.2.3 Efficacy and mechanisms of HBOT in treating RIDs with comorbidities

Behçet's disease, an autoinflammatory vasculitis, can affect cutaneous vessels, leading to oral and genital ulcers, as well as inner ear vasculature, resulting in sensorineural hearing loss (SNHL). A case report described a 21-year-old male Behçet's patient with sudden severe bilateral SNHL whose hearing thresholds improved by 20 dB following corticosteroid therapy and 15 consecutive days of HBOT (44). Although limited literature exists on HBOT for SNHL secondary to Behçet's disease, the American Academy of Otolaryngology–Head and Neck Surgery endorses HBOT as first-line therapy for idiopathic SNHL (46). A meta-analysis also found that adding HBOT to standard pharmacotherapy significantly enhances complete hearing recovery, any degree of hearing recovery, and absolute hearing gain in SNHL patients, particularly those receiving at least 1,200 min of HBOT (47). The mechanism of HBOT in SNHL involves vasodilation in the organ of Corti and other inner ear structures, counteracting vascular damage and oxidative stress (48). It also effectively reduces endolymphatic hydrops induced by bacterial or viral infections (49), thereby improving hearing.

Additionally, HBOT has been reported as adjunctive therapy for systemic lupus erythematosus (SLE) patients with acute macular neuroretinopathy (AMN) (50). Animal studies have demonstrated that HBOT combined with reduces inflammation in rheumatoid arthritis (RA)-associated interstitial lung disease in rat models (51). Furthermore, successful HBOT treatment of pneumatosis cystoides intestinalis in a granulomatosis with polyangiitis patient has been documented, with no complications upon discharge (52).

### 3.3 Limitations

This study has several limitations. The literature evaluation indicated that most included studies were case reports or case series, with few controlled trials and generally low-quality evidence. Furthermore, hyperbaric oxygen therapy remains relatively novel in rheumatology, which limited the number of available studies employing consistent methodologies despite a comprehensive search. This methodological heterogeneity may compromise the reliability of the conclusions. Future large-scale randomized controlled trials from multiple countries are required to establish more robust evidence.

## 4 Summary

HBOT demonstrated remission rates of 87.5%−100% in the treatment of RIDs complicated by skin ulcers and FMS, with enhanced efficacy observed after a full treatment course. HBOT also exhibited favorable therapeutic effects in RIDs associated with SNHL and AMN. As a well-established and non-invasive intervention with minimal adverse effects and significant efficacy, HBOT holds promising potential in the management of RIDs, serving as a novel non-pharmacological option for adjunctive treatment of skin ulcers, joint and muscle pain, and other symptoms. However, most existing studies are limited to case reports or retrospective analyses, and apart from research on HBOT for FMS, the available literature primarily consists of small-sample RCTs. Further validation through multicenter, large-scale RCTs is warranted to evaluate the efficacy and safety of HBOT at different ATA and treatment durations for RIDs.
